# Dosimetric quality of HyperArc in boost radiotherapy for single glioblastoma: comparison with CyberKnife and manual VMAT

**DOI:** 10.1186/s13014-022-02150-y

**Published:** 2023-01-10

**Authors:** Mingyuan Pan, Wenqian Xu, Lei Sun, Chaozhuang Wang, Shengnan Dong, Yun Guan, Jun Yang, Enmin Wang

**Affiliations:** 1 CyberKnife Center, Department of Neurosurgery, Huashan Hospital, Fudan University, Shanghai, China; 2 Neurosurgical Institute, Fudan University, Shanghai, China; 3 Shanghai Clinical Medical Center of Neurosurgery, Shanghai Key Laboratory of Brain Function and Restoration and Neural Regeneration, 12 Wulumuqi Road (M), Shanghai, 200040 China; 4 Radonc Department, Foshan Chancheng Hospital, 3 Sanyou Road, Foshan, 528000 Guangdong China

**Keywords:** Stereotactic radiotherapy, Dosimetry, CyberKnife, HyperArc, Glioblastoma

## Abstract

**Background:**

Stereotactic radiotherapy (SRT) and hypo-fractionated radiotherapy are feasible treatment options for single glioblastoma multiforme when combined with conventional radiotherapy or delivered alone. HyperArc (HA), a novel linac-based method with 4 noncoplanar arcs, has been introduced into stereotactic radiosurgery (SRS) for single and multiple metastases. In this study, we compared the dosimetric quality of HyperArc with the well-established CyberKnife (CK) and conventional VMAT methods of SRT for a single, large target.

**Methods:**

Sixteen patients treated in our center with their clinical CK plans were enrolled, and the linac-based plans were designed in silico. From the aspect of normal tissue protection and treatment efficacy, we compared the conformity index (CI), gradient index (GI), homogeneity index (HI), dose distribution in planning target volume, dose in the normal brain tissue, and mean dose of several organs at risk (OARs). All of the data were evaluated with nonparametric Kruskal‒Wallis tests. We further investigated the relationship of the dose distribution with the tumor volume and its location.

**Results:**

The results showed that with a higher CI (0.94 ± 0.03) and lower GI (2.57 ± 0.53), the HA plans generated a lower dose to the OARs and the normal tissue. Meanwhile, the CK plans achieved a higher HI (0.35 ± 0.10) and generated a higher dose inside the tumor. Although manual VMAT showed slight improvement in dose quality and less monitoring units (2083 ± 225), HA can save half of the delivery time of CK (37 minutes) on average.

**Conclusion:**

HA plans have higher conformity and spare OARs with lower normal tissue irradiation, while CK plans achieve a higher mean dose in tumors. HA with 4 arcs is sufficient in dosimetric quality for a single tumor with great convenience in planning and treatment processes compared with conventional VMAT. The tumor size and location are factors to be considered when selecting treatment equipment.

## Background

Central nervous system (CNS) tumors are lethal illnesses that account for 1.6% of cancer cases and have a rather high mortality rate of 2.5% [1]. They can affect CNS function and be life-threatening due to their aggressive growth, invasiveness and mass effect. GBM is the most common malignant tumor in the CNS (14.5% of all CNS tumors and 48.6% of malignant tumors in the CNS) [2]. Radiation therapy is one of the primary modalities for treating malignant brain tumors, with a 1.3-fold increase in the median lifespan associated with a curative-intention dose compared to no radiotherapy [3]. However, GBM tumors are relatively resistant to conventional radiotherapy and are typically treated with a definitive dose of 60 Gy in 30 fractions.

Given the poor prognosis, other treatment regimens, such as SRT, have been explored. SRT utilizes highly precise radiation techniques to deliver a high biological equivalent ablative prescription dose to the tumor while minimizing the dose to the adjacent normal tissues [4]. This therapy remains controversial, as GBM is a highly infiltrative disease. ASTRO reports in 2015 showed that there was insufficient evidence regarding the benefit or harm of using a radiosurgery boost in terms of overall survival [5]. However, regarding reirradiation for progressive and recurrent glioblastoma, and based on recently reviewed data, hypo-fractionated radiotherapy could improve local control [6].

Several trials have evaluated the use of hypo-fractionated radiotherapy in newly diagnosed GBM patients [7]. Roa et al. [8] reported that 40 Gy/15 fx HFRT improved overall survival (OS) compared with 60 Gy/30 fx conventional RT (5.6 months vs. 5.1 months, P < 0.05) in a randomized trial in 2004. Roa et al. [9] also reported an OS of 7.9 months using 25 Gy/5 fx HFRT in elderly and/or frail GMB patients in 2015. Another randomized phase 3 trial compared temozolomide, 60 Gy/30 fx standard RT and 34 Gy/10 fx HFRT. For patients older than 70 years, survival was more favorable with HFRT than standard RT, and HFRT also resulted in lower toxicity and a shorter treatment duration.

Since gamma knife is an invasive frame-based SRS technology, a frame-less robotic radio-surgical device called CyberKnife (Accuray, Sunnyvale, CA) has been developed [10]. In 1996, Murphy and Cox described the accuracy of the first-generation CyberKnife and found it comparable to that of existing frame-based systems. Since then, CyberKnife has been proven to be an alternative method for intracranial SRT, and its potential for treating GBM has also been reported [11, 12]. We retrospectively analyzed our patients with High Grade Gliomas (HGG) undergoing HFRT and found favorable outcomes and acceptable toxicity in our center [13].

Recently, technical advances, such as cone-beam CT imaging, robotic couches, improved patient positioning systems and flattening filter-free beams, have made linac-based SRT more efficient and patient-friendly than traditional linac systems [14]. Unlike conventional VMAT methods, HyperArc (Varian, Palo Alto, CA) uses both coplanar and noncoplanar arcs to treat single or multiple intracranial targets with one isocenter, and the treatment can be optimized and executed by the machine automatically. It can irradiate the targets while keeping a sharp dose "fall off" outside the target to minimize the dose to certain sensitive organs [15].

Several studies have compared the dosimetry of linac-based SRT techniques with that of CyberKnife or manual VMAT in single or multiple brain metastases. Ohira suggested that HyperArc provided significantly higher conformity and rapid dose fall-off compared with conventional VMAT planning, and this novel method also improved the treatment efficiency [16, 17]. Ruggieri also found that HA plans assured a higher CI and a lower GI than standard multiple-isocenter VMAT plans [18]. Experience and clinical results also showed a promising future for HyperArc in the application of SRT [19]. Both Kadoya and Slosarek made dosimetric comparisons of CyberKnife and HyperArc to find better homogeneity and conformity while lowering GI with HyperArc [20, 21].

However, all of the research listed above compared single or multiple brain metastases. Here, we aimed to evaluate the dosimetric quality of HyperArc in comparison with other techniques for providing boost therapy for solitary GBM lesions with large volumes.

## Methods

### Patient selection

A cohort of 16 patients (aged 6–72 years; 9 women and 7 men) previously treated from 2018 to 2021 in our center were enrolled in this retrospective in silico study. The study was approved by our institutional review board (IRB), and all of the patients signed informed consent forms prior to treatment. All of the patients were diagnosed with solitary tumors with a mean volume of 25.3 cm^3^ (range 4.5–78.4 cm^3^), and the demographics of these patients are listed in Table 1.

**Table 1:** Patient characteristics and volume and position of tumors

Number	Gender	Age (years)	Tumor volume (cc)	Tumor position
1	F	6	4.5	Brain stem
2	M	66	10.8	Temporal lobe (R)
3	F	72	5	Temporal lobe (R)
4	M	66	20.8	Frontal lobe (M)
5	F	41	35.5	Temporal lobe (L)
6	M	66	35.6	Occipital lobe (R)
7	F	65	12.3	Occipital lobe (L)
8	M	27	6.7	Temporal lobe (L)
9	F	23	12.7	Frontal lobe (M)
10	M	58	40.4	Frontal lobe (R)
11	M	42	3.7	Frontal lobe (R)
12	M	40	42.5	Temporal lobe (L)
13	F	26	35.2	Temporal lobe (R)
14	F	48	26.5	Occipital lobe (L)
15	F	61	33.6	Parietal lobe (R)
16	F	71	78.4	Temporal lobe (L)

Patients were immobilized with a customized thermoplastic mask and were simulated using computed tomography (CT, Toshiba 64 Slice, Japan) with iohexol contrast (Omnipaque, Amersham, UK) from the top of the skull to the chin with a slice thickness of 1 mm and then with thin-slice (2 mm) 3.0 T MRI (GE 750 W, USA) with dimeglumine gadopentetate contrast (dimeglumine gadopentetate injection, Beijing, China). CT and MRI scans were then registered in the treatment planning system (TPS) (Precision, version 1.1, Accuray, Sunnyvale, CA) to contour the target and other organs at risk. The prescription dose was 30 Gy/5 fx with 95% volume of the PTV covered. The prescription isodose line was set to 70%-75% in the CK plan, and the D_max_ was limited to 150% in the linac-based plan.

### Treatment planning

#### Cyberknife plan

CT and MRI images were loaded in the Multiplan system (Ver 4.6, Accuray Inc., Sunnyvale, USA) to contour the target and normal structures. Gross tumor volume (GTV) was defined as the gadolinium-enhanced tumor on the T1-weighted MRI. The clinical target volume (CTV) was considered equal to the GTV. The planning target volume (PTV) was a uniform 2 mm expansion of the CTV, and FLAIR abnormalities were not included in the treatment volume.

All of the plans were optimized in sequential mode using the Ray Tracing algorithm by experienced physicists in our center and were approved by the physicians prior to the clinical treatment. The dose grid size for the calculation was set at high resolution. 6D skull tracking and the full path were used for treatment. Both fixed size cones (12 fixed apertures with diameters ranging from 5 to 60 mm) and iris collimators (12 variable apertures with diameters ranging from 5 to 60 mm) were used for the collimation of the photon beams (6 MV). The number of nodes where the robot arm stops and delivers the irradiation beams ranges from 82 to 112, and the number of irradiation beams ranges from 134 to 234. The maximum MU of each node was limited to 500 MUs in 5 fractions. All of the treatment times were kept to less than 40 min for clinical convenience.

The D_max_ of the optical apparatus (eyeballs, optical nerve, chiasma) should be less than 25 Gy/5 fx. The D_0.5cc_ of the brainstem should be less than 23 Gy/5 fx [22]. Several shells or other control regions were made to increase the dose within the target as well as accelerate the dose fall-off in the surrounding tissues.

#### HyperArc plan

These cases were replanned in the Eclipse System (Version 15.5, Varian Medical Systems, Palo Alto) using the same DICOM images, prescription, OAR contours and dose constraints.

HyperArc is a linac-based technique operated on a TrueBeam linear accelerator equipped with a 2.5-mm leaf-width Multi-leaf Collimator (MLC). Unlike the conventional coplanar VMAT, it uses one fixed geometry setup with a maximum of 4 arcs, including 3 noncoplanar arcs with one isocenter. Furthermore, the treatment process, from the selection of collimator angles to the treatment delivery, is automatically designed according to the position and number of targets [23]. Meanwhile, this technique can perform collision checks and prevention with a virtual dry run mechanism.

**Fig. 1:**
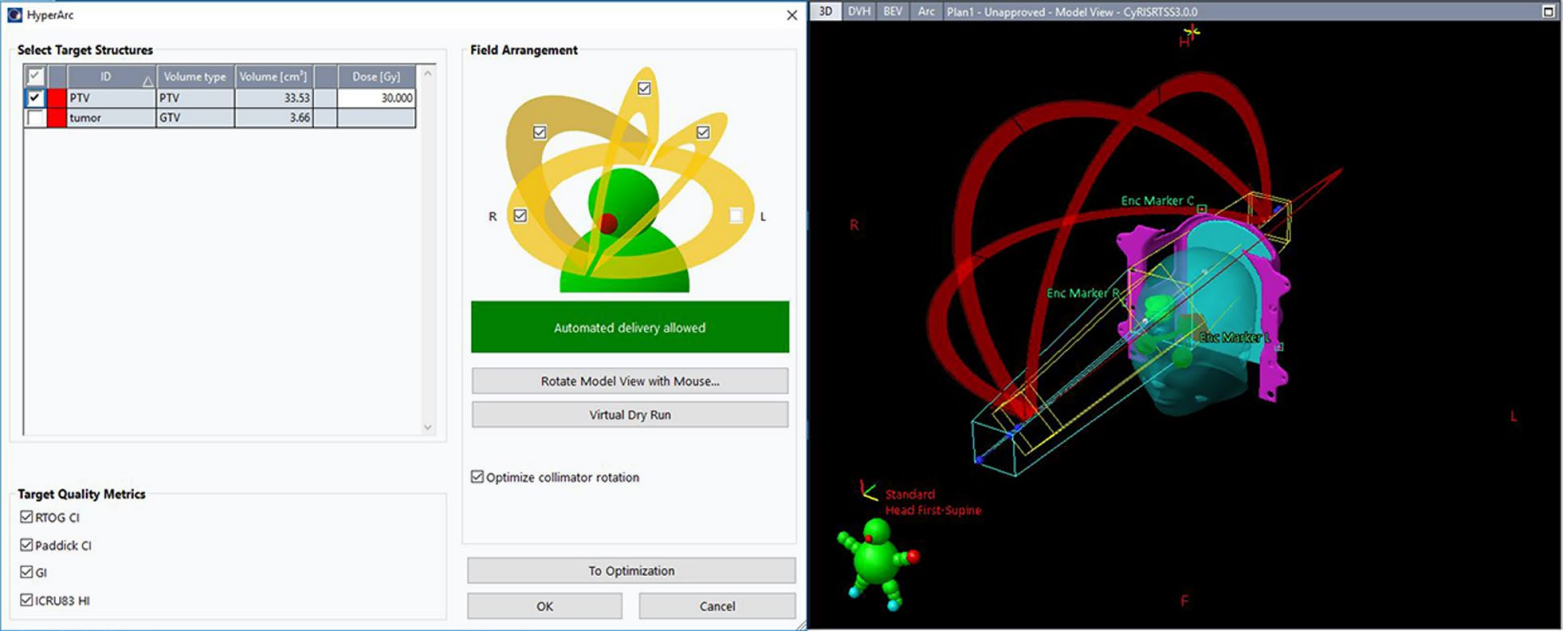
A screen shot of users’ interface for the HA plans in Eclipse. With dry run, HyperArc can perform collision check to select partial arcs

In our study, the structures of the Encompass (Qfix, Avondale, USA) SRS immobilization system were inserted into the CT image. All four arcs were used with an automatically selected isocenter, optimized collimator rotation and jaw tracking, as demonstrated in Fig. 1. A virtual dry run was played in the TPS to avoid potential collisions. As a hyperfractioned radiotherapy, flattening filter-free (FFF) beams with a 6-MV photon beam at a maximum dose rate of 1400 MU per minute were used. Even though the FFF technique can cause dose distribution heterogeneity, considering that a hot spot inside the target area is usually preferred in SRT and that the FFF technique can reduce the scatter dose outside of the treatment field, we consider FFF to be favorable for intracranial SRT [24].

In the optimization by the optimizer engine PO (Varian, v.15.5.12), a normal tissue objective SRS-NTO is automatically set for HA plans. In this case, SRS-NTO reduces the dose to healthy surrounding brain tissue and maximizes the steepness of the dose gradient. The dose grid resolution was set as 1.25 mm in the AXB algorithm (Varian, v.15.5.12), with all of the structures assigned to the proper mass and electron density model.

#### Manual VMAT plan

**Fig. 2:**
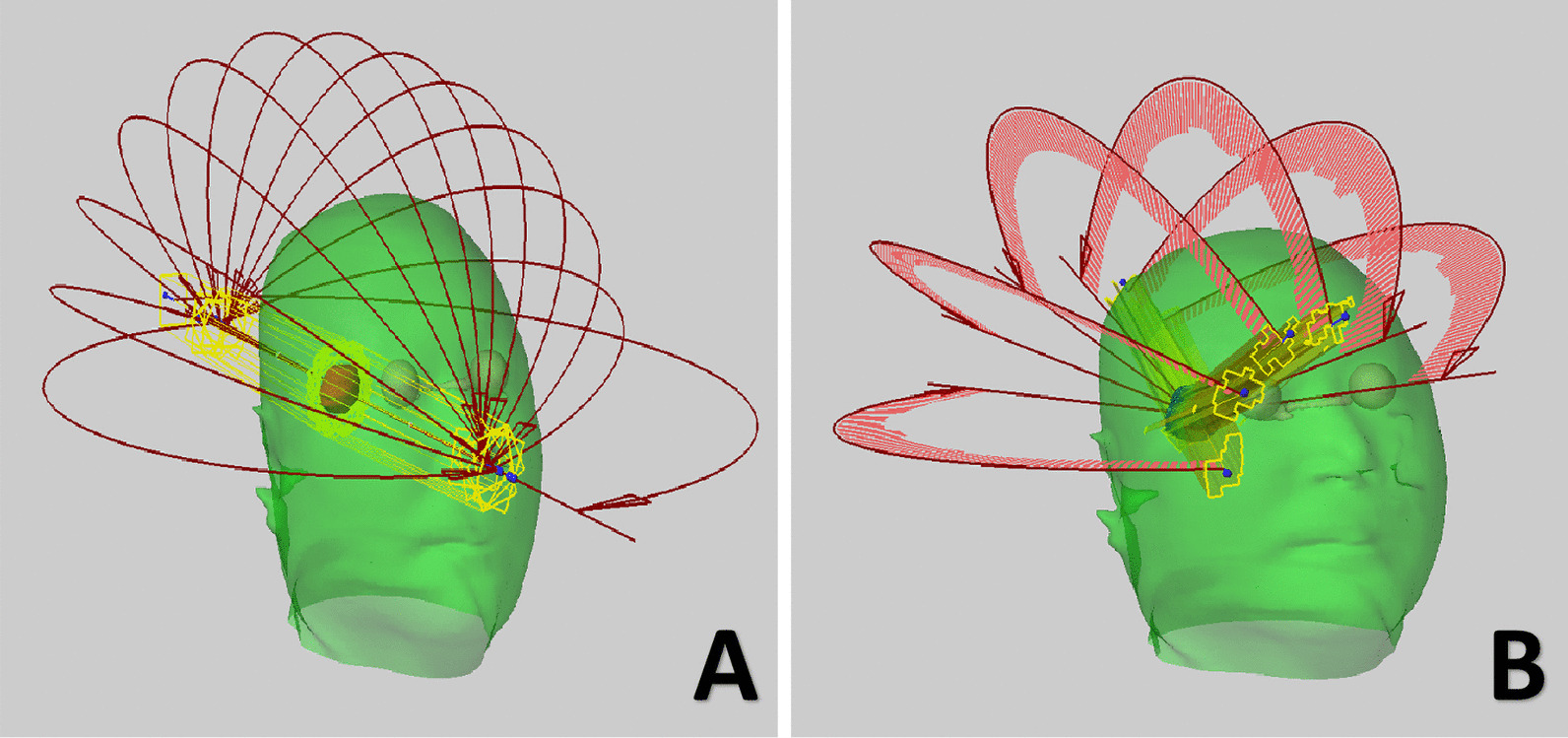
A ‘SRS Helmet’ template was inserted for manual VMAT planing in (**A**). And 6 arcs with 150 degree of length was selected for optimization. The results and projection of MLC shape was shown (**B**)

All linac-based plans were redesigned based on TrueBeam with HDMLC. A model called the ‘SRS Helmet’ was inserted, as illustrated in Fig. 2. The common isocenter was automatically located at the centroid of the tumor, while the center of the infratentorial tumor was shifted coronal to avoid potential collisions based on our own experience. The jaws of the collimator were adjusted to be parallel to the tumor on the Y axis. By observing the relative position and target, 5–8 partial arcs were selected based on the principle of sparing more normal tissue, covering the whole tumor and avoiding the OARs. In addition, coplanar arcs are necessary when dealing with complicated shapes. The design is summarized in Table 2. The beam setup was the same as the HyperArc planning, and many control rings were created to escalate the dose around the tumor in the absence of the function of SRS-NTO and ALDO. In the dose calculation process, the resolution and algorithm remained the same as those of the automatic approach.

**Table 2:** Planning design for manual VMAT

Number	Number of arcs	Arc length (couch angle)
1	5	360 (0 0 0) 180 (90 90)
2	6	140 (0 30 60 90 300 330)
3	5	180 (0 30 60 90 300)
4	6	180 (15 45 75 285 315 345)
5	6	180 (0 45 75 285 315 345)
6	7	150 (0 30 45 75 285 285 315)
7	6	180 (0 0 30 60 90 300 330)
8	6	180 (285 300 315 330) 200 (0 0)
9	6	360 (0) 120 (30 60 90 300 330)
10	6	180 (0 0 30 60 90 330)
11	5	180 (0 15 45 75 285)
12	8	180 (0 45 75 90 285 300 330 345)
13	6	140 (0 30 60 90 300 330)
14	6	180 (0 30 60 90 300 330)
15	7	180 (0 0 30 60 90 300 330)
16	6	180 (0 0 75 285 315 345)

### Dosimetric quality assessment

All plans were exported in DICOM format and imported into Velocity4.0 (Varian Medical Systems, Palo Alto, CA) for comparison purposes. The plan assessments of the HyperArc and CyberKnife were performed by evaluating the CI, HI, and GI.

CI defines how precisely the prescription dose distribution matches the target. However, the RTOG-CI has been considered less accurate because the target volume (TV) and prescription isodose volume (PIV) are not necessarily concentric and symmetrical. In this study, we chose the Paddick conformity index (PCI) = (TTV*TTV)/(TV*PIV), where TTV represents the volume of the target covered by the prescription isodose [25] .

HI is used to define the homogeneity of the dose coverage and is calculated as: HI = (D_2%_−D_98%_)/Dp.

GI is the ratio of the irradiated volume enclosed by 50% of the prescription dose divided by the volume of PIV, GI = PIV_50%_/PIV. GI describes the steepness of the dose gradient from PIV to the surrounding tissue. [26]

In addition, cold spots, mean dose and volume covered by 95% of the prescription dose of the PTV were observed to compare the target coverage.

Since 12 Gy in single fraction SRS can cause brain tissue necrosis, and we were using 5 fraction SRS regimens, we converted its biological effective dose (BED) to 23 Gy in 5 fractions as a higher dose region in NT using the LQ model [27]. In addition, the effect of sparing normal surrounding brain tissue was also compared using the volume of a lower dose region of 10 Gy, as well as the mean dose in NT.

All of the OARs for the dose constraint are presented in “Cyberknife plan” section. Typically, the optical apparatus and brainstem are considered serial-type organs and are usually constrained by the “maximum dose”. However, the maximum dose was variable in the calculation resolution in our cases. As such, in our study, we compared the mean dose of each OAR instead.

### Statistical analysis

Statistical analysis was performed using SPSS (version 22.0, IBM) and MATLAB (version 2016, MathWorks). The mean and standard deviation were computed for each dosimetric parameter. The significance of the difference for all of the plans was evaluated using the p value derived from the nonparametric Kruskal‒Wallis test with a threshold of 0.05. Spearman bivariate correlation analysis was also performed to determine the relationship between each parameter and the tumor volume.

## Results

All of the dosimetric parameters mentioned above are listed in Table 3. There was a significant dosimetric difference between all of the irradiation techniques in terms of tumor irradiation, with a less significant difference for sparing the normal brain tissue and OARs.

**Table 3:** Dosimetric parameters for CyberKnife and HyperArc plans of 16 patients

Structure	Dosimetric parameters	All (n = 16)						
		CyberKnife plan		HyperArc plan		M-VMAT plan		p value
		Mean	Std	Mean	Std	Mean	Std	
Tumor	CI	0.85	0.05	0.94	0.03	0.95	0.02	< 0.05
	HI	0.42	0.04	0.35	0.10	0.38	0.06	0.154
	GI	3.29	0.35	2.57	0.53	2.75	0.48	< 0.05
	D_mean_	36.36	0.78	34.92	1.30	36.22	0.97	< 0.05
	D_min_	24.95	2.64	26.74	2.43	27.95	1.38	< 0.05
	V_95%_	99.21	0.44	99.77	0.26	99.85	0.12	< 0.05
Normal tissue	V_23Gy_	22.50	6.85	11.84	6.69	15.69	7.37	0.144
	V_10Gy_	110.57	71.29	69.82	40.37	77.89	34.40	0.282
	D_mean_	3.90	1.71	3.02	1.19	3.54	1.32	0.210
Organs at risk	D_mean_Brainstem_	4.39	4.28	3.50	3.75	3.28	3.95	0.128
	D_mean_Chiasma_	4.53	2.83	3.08	1.55	2.89	2.84	0.189
	D_mean_EyeL_	1.77	2.15	1.04	0.80	1.18	1.98	0.325
	D_mean_EyeR_	1.37	0.83	0.93	0.66	0.95	0.50	0.160
	D_mean_OptNerveL_	3.06	3.90	1.87	1.71	1.99	1.80	0.054
	D_mean_OptNerveR_	2.74	1.69	1.78	1.12	1.76	1.01	0.422

All the unit for Dose are Gy, the unit for volume are cm^3^

**Fig. 3:**
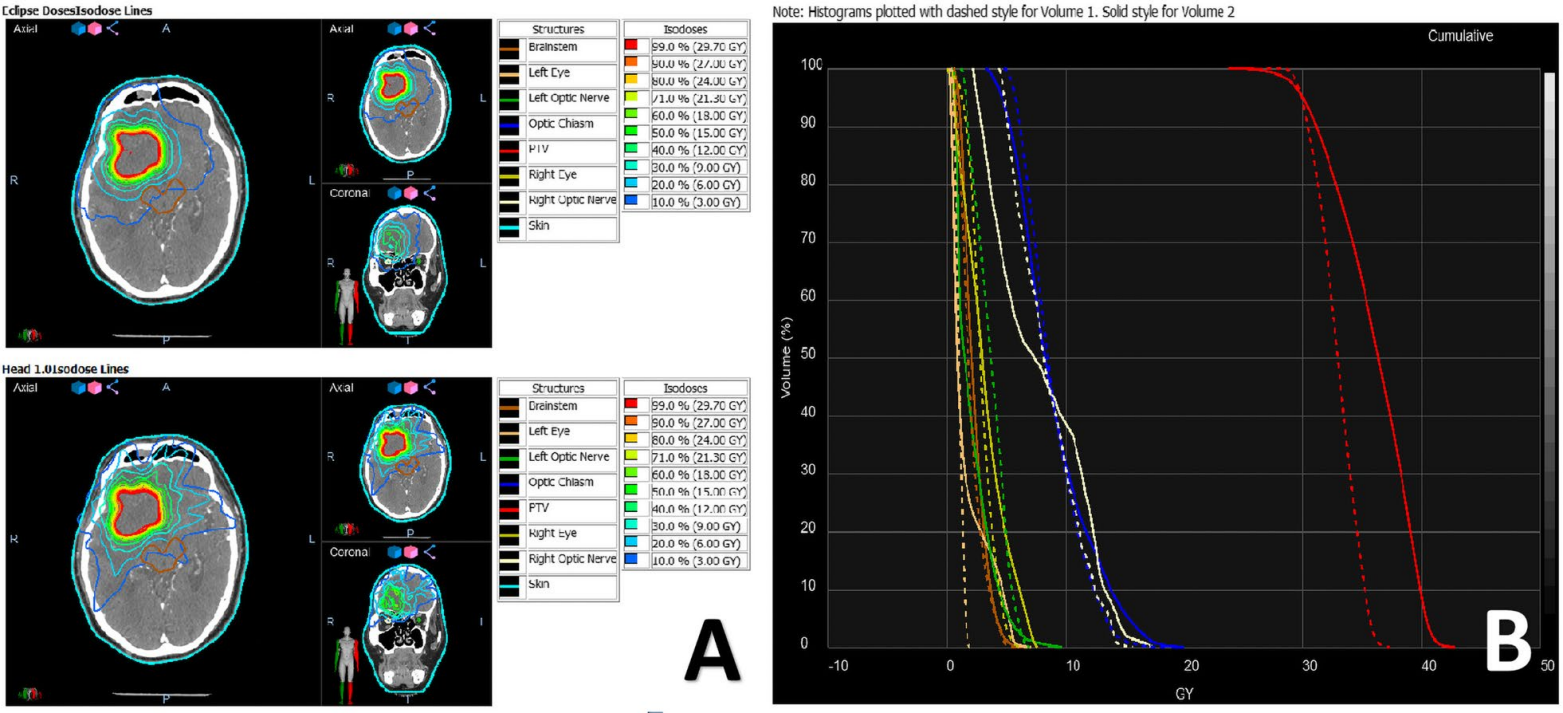
Comparison of isodose distribution and DVH of CK and HA of patients via Velocity, the upper and lower in **A** represented HA and CK plans respectively. And dashed line and solid line in **B** represented HA and CK plans

Figure 3 shows the comparison of the isodose distribution and dose-volume histogram between CK and HA plans for one patient via Velocity. With a lower GI and higher CI, the isodose spread as a radial pattern outside the tumor volume in the CK plans (left lower corner), where the normal tissue received 9 Gy, a much higher dose than in the HA plans (left upper corner). Meanwhile, the isodose lines from 30 to 12 Gy of the HA plans are more compact. As illustrated in the histogram (right), the solid red line representing the PTV of the CK plans covered a larger space under the line than the dashed line, indicating a higher irradiation dose in the CK plan. However, nearly all OARs received a higher maximum dose from the CK plans in the histogram.

**Fig. 4:**
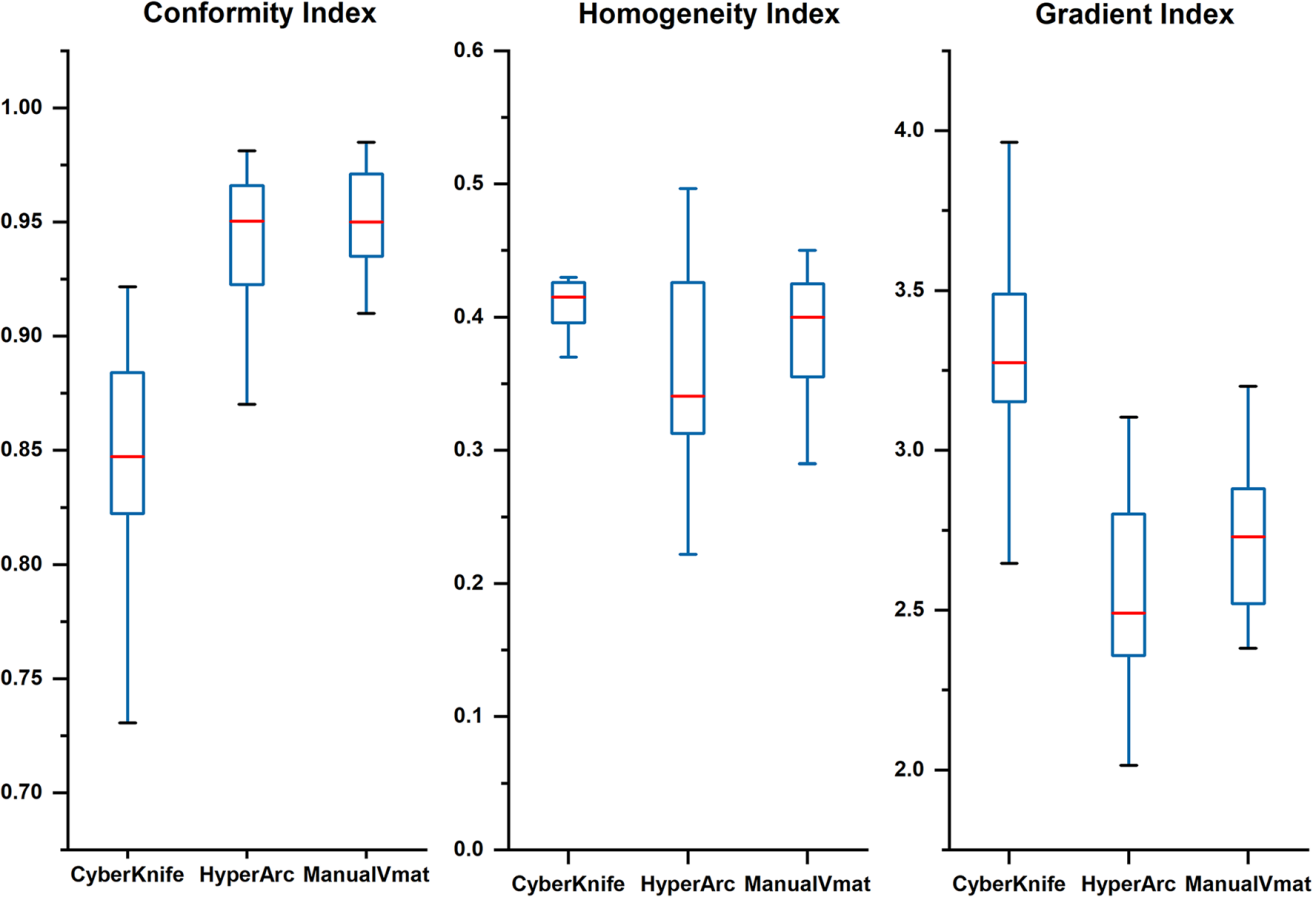
Boxplots of dosimetric parameters of CI, HI, and GI for tumor volume of CK, HA and manual VMAT plans. And the red line showed the median value indicating that HyperArc had a higher CI and lower GI than Cyberknife and had no statistic significant difference with manual VMAT

Figure 4 shows the results of the dosimetric parameters with regard to CI (0.85 ± 0.05, 0.94 ± 0.03, and 0.95 ± 0.02 for CK, HA, and VMAT, respectively), HI (0.42 ± 0.04, 0.35 ± 0.10, and 0.38 ± 0.06 for CK, HA, and VMAT, respectively) and GI (3.29 ± 0.35, 2.57 ± 0.53, and 2.75 ± 0.48 for CK, HA, and VMAT, respectively). A significant difference was observed in that the linac-based plans had a tighter conformity (p < 0.05) and a sharper dose fall-off (p < 0.05) than the CK plans. Meanwhile, the differences in the performance of HA and Manual VMAT in terms of CI (p = 0.53) and GI (p = 0.40) were not significant.

**Fig. 5:**
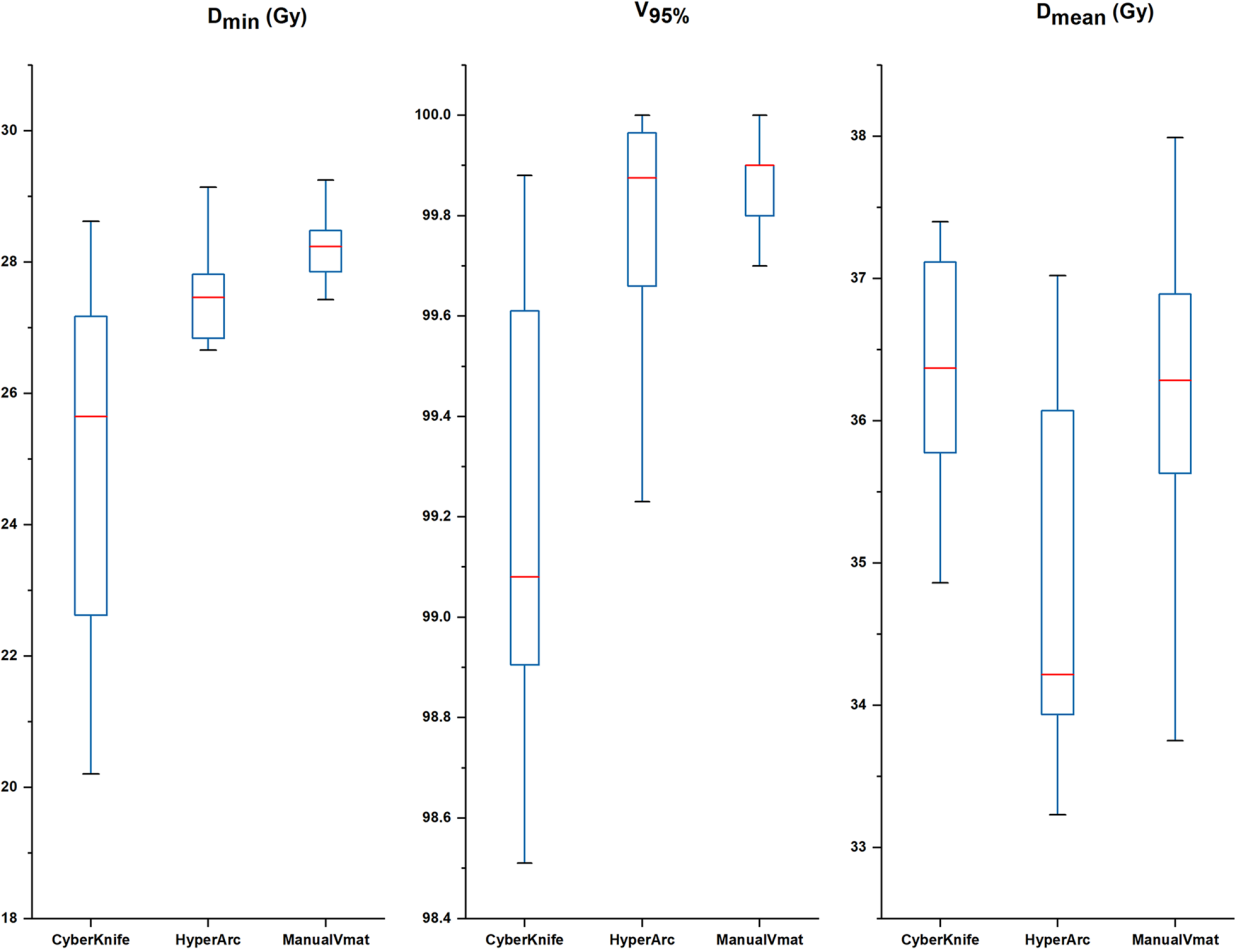
Boxplots of dosimetric parameters of D_min_, V95%, and D_mean_ for CK, HA and manual VMAT plans. The results shows that CK plans had more cold spots, while achieved higher mean dose irradiation in target

Concerning the target coverage, Fig. 5 compares the D_min_ (24.95 ± 2.64, 26.74 ± 2.43, and 27.95 ± 1.38 for CK, HA, and VMAT, respectively), V_95%_ (99.21 ± 0.44, 99.77 ± 0.26, and 99.85 ± 0.12 for CK, HA, and VMAT, respectively) and D_mean_ (3.90 ± 1.71, 3.02 ± 1.19, and 3.04 ± 1.32 for CK, HA, and VMAT, respectively) of the PTV. All of the results showed a significant difference, indicating that conventional VMAT achieved relatively better performance in target coverage. Although the CK plans had a lower minimum dose (p < 0.05) and coverage rate (p < 0.05), their mean dose was much larger than that of the HA plans (p < 0.05) and was not different from that of the manual plans (p = 0.45).

**Fig. 6:**
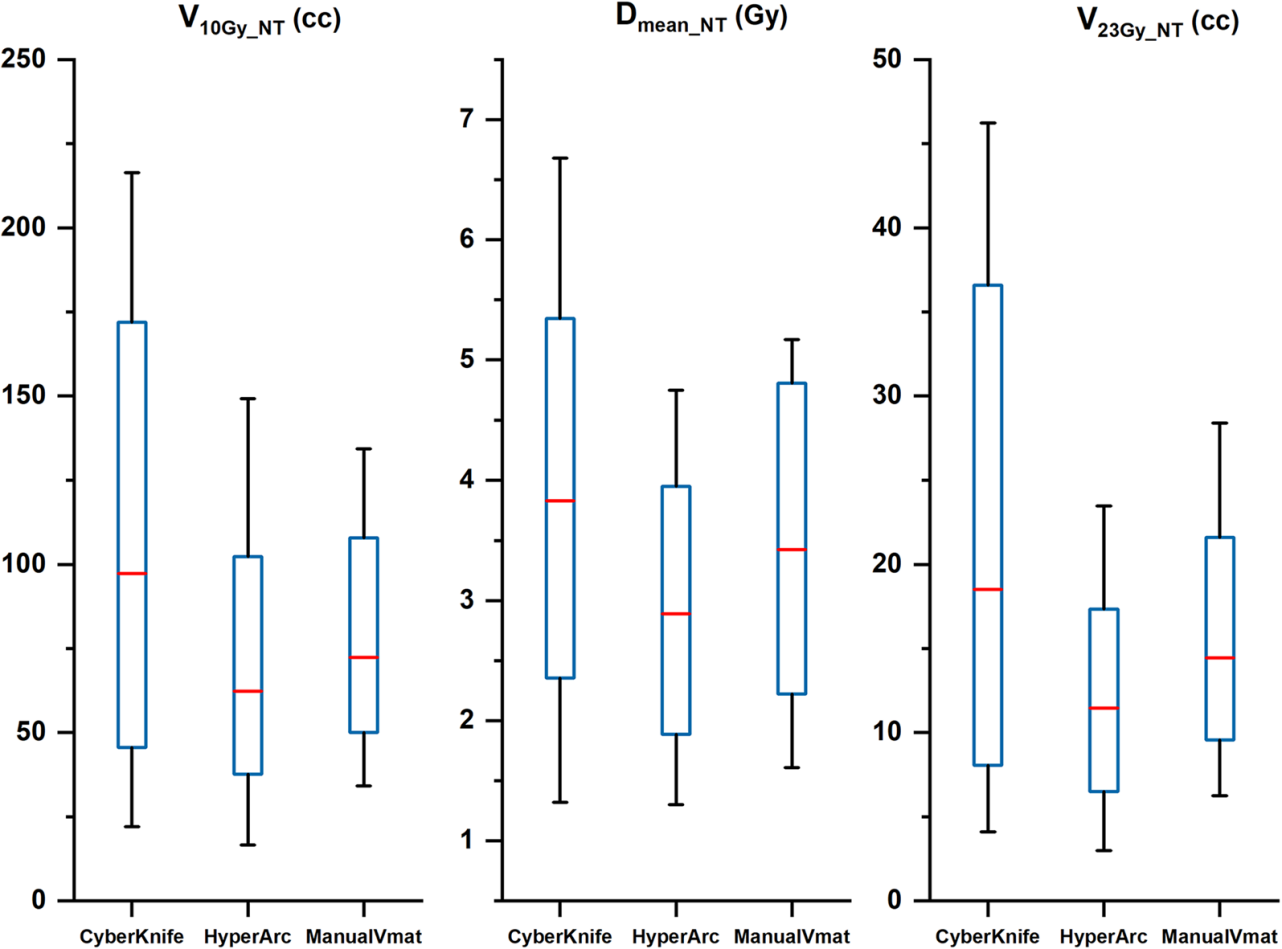
Boxplots of dosimetric parameters of mean dose, volume received 10 Gy and 23 Gy in normal tissue (Brain cropped tumor) of CK and HA plans. It indicated that normal tissue in HA plans receive a lower dose

In terms of sparing normal tissue, it can be seen in Fig. 6 that the linac-based plan achieved a relatively lower dose distribution in the whole brain than CK, with smaller volumes of 23 Gy (12.13 ± 6.85, 11.84 ± 6.69, and11.69 ± 7.37 for CK, HA, and VMAT, respectively) and 10 Gy (110.57 ± 71.29, 69.82 ± 40.37, and 71.76 ± 134.40 for CK, HA, and VMAT, respectively). The performance of HA and Manual VMAT in protecting the normal brain showed no significant difference.

**Fig. 7:**
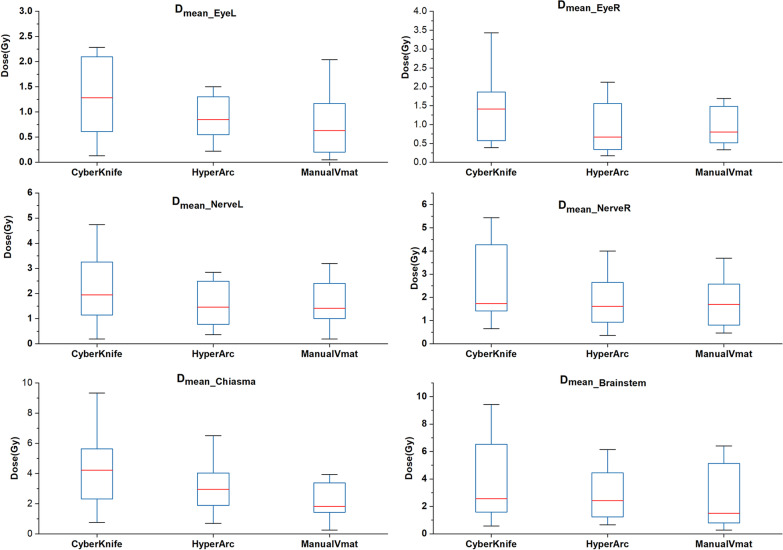
Boxplots of dosimetric parameters of mean dose in chiasma, left eyeball, right eyeball, brainstem, left optical nerve, and right optical nerve of CK and HA plans. The results showed that linac-based plan performs better in protecting OARs

There were also no statistically significant differences in the comparison of the D_mean_ of OARs, as Fig. 7 illustrates, where the CK plans had a higher mean dose for all of the critical organs. HA had a lower D_mean_ in the left eyeballs (1.77 ± 2.15, 1.04 ± 0.80, and 1.18 ± 1.98 for CK, HA, and VMAT, respectively), right eyeballs (1.37 ± 0.83, 0.93 ± 0.66, and0.95 ± 0.50 for CK, HA, and VMAT, respectively) and left optic nerves (3.06 ± 3.90, 1.87 ± 1.71, and 1.99 ± 1.80 for CK, HA, and VMAT, respectively), while conventional VMAT had a lower D_mean_ in the right optic nerves (2.74 ± 1.69, 1.78 ± 1.12, and 1.76 ± 1.01 for CK, HA, and VMAT, respectively), chiasma (4.53 ± 2.83, 3.08 ± 1.55, and 2.89 ± 2.84 for CK, HA, and VMAT, respectively), and brainstem (4.39 ± 4.28, 3.50 ± 3.75, and 3.28 ± 3.95 for CK, HA, and VMAT, respectively).

## Discussion

This study conducted a comprehensive comparison of the dosimetric quality of three irradiation techniques for boosting therapy for GBM, including their performance in dose conformity, homogeneity, gradient, target irradiation, and ability to spare the normal tissue and OARS. The results showed that the linac-based plan performed better in dosimetric parameters with a higher Paddick CI and lower GI, while HA with 4 arcs and conventional VMAT showed no statistically significant differences.

For the dose distribution to the target, with a lower CI, the CK plan had more cold points than the others. However, with a larger HI, its mean dose and maximum were much larger than those of the HA plan. The reason might be that HA with an HDMLC of 0.25 mm tends to fit the boundary of the target better, and the cone beams of CK are more difficult to control in terms of the inner dose region and are more likely to deliver the dose to the target repeatedly. In contrast, manual VMAT with HDMLC showed a high conformity; nevertheless, its D_mean_ in the PTV was as high as that of the CK plan. The reason for this might be the difference in optimization functions as well as the automatic collimator optimization. With the functions of CAO, ALDO and SRS-NTO, the HA plans were optimized with no more constraints or objectives to target in the first trial. However, more objectives, including the gEUD functions and controlled structures, were introduced in the manual plan to satisfy the dose constraints to the PTV.

**Fig. 8:**
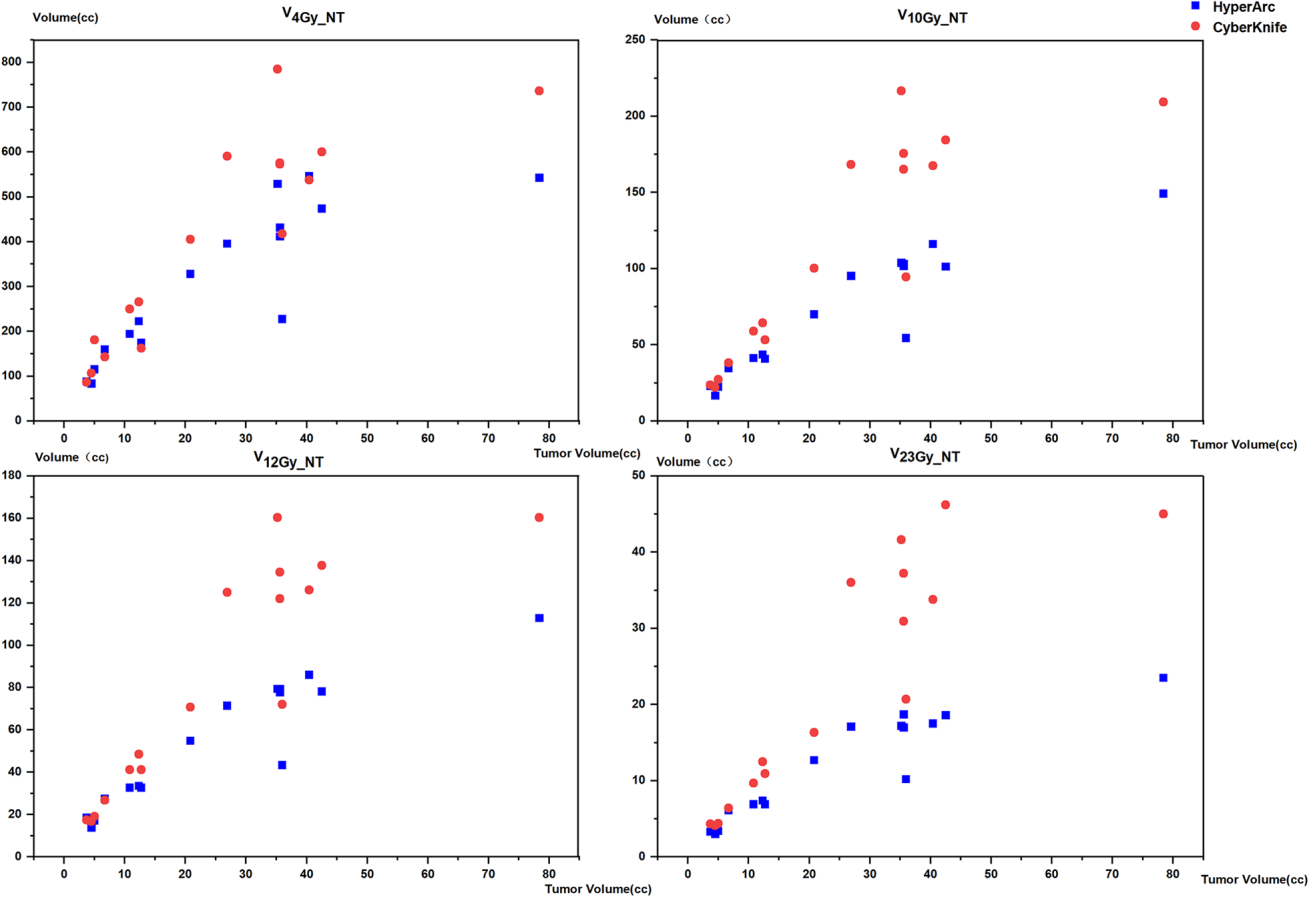
Scatter diagram of HA and CK plans in V_4Gy_, V_10Gy_, V_12Gy_, V_23Gy_ with tumor volume as y axis showing that parameters have a correlation with tumor volumes. Blue spots represent the HA plans and red spots represent the CK plans. And by a spearman bivariate correlate analysis, the D-value of two parameters with tumor volume are 0.55, 0.81, 0.82, 0.90, respectively

Although the results for the comparison of protecting normal tissue failed to pass the Kruskal‒Wallis test with a threshold of 0.05, CK achieved a significantly higher GI than HA. The results obtained in our study differed from those reported in the literature for multiple metastases [21]. Considering the situation of a much larger tumor volume, we evaluated the relationship between each parameter’s difference value (D-value) of CK and HA and their tumor volume by Spearman bivariate correlation analysis. All of the parameters of CK and HA passed the double-paired significance t-test with a threshold of 0.05. With a Spearman value of 0.70 for GI showing a certain relationship with the tumor volume, the scatter diagrams of V_4Gy_, V_10Gy_, V_12Gy_, and V_23Gy_ in the normal brain for CK and HA are shown in Fig. 8, with correlation coefficients of 0.55, 0.81, 0.82, and 0.90, respectively. It can be seen that with an increase in the target volume, the gap of HA and CK for sparing the normal brain tissue would be enlarged, especially in the moderate- and high-dose regions outside the PTV.

In addition, when the tumor volume was as small as that of a single metastasis in one case, CK showed a better GI of 3.67 than HA (4.01). These results are consistent with the conclusion in Kadoya’s research [20], which gives clinicians and medical physicists a different perspective when selecting the appropriate medical equipment for applying SRT to different sizes of GBM.

**Fig. 9:**
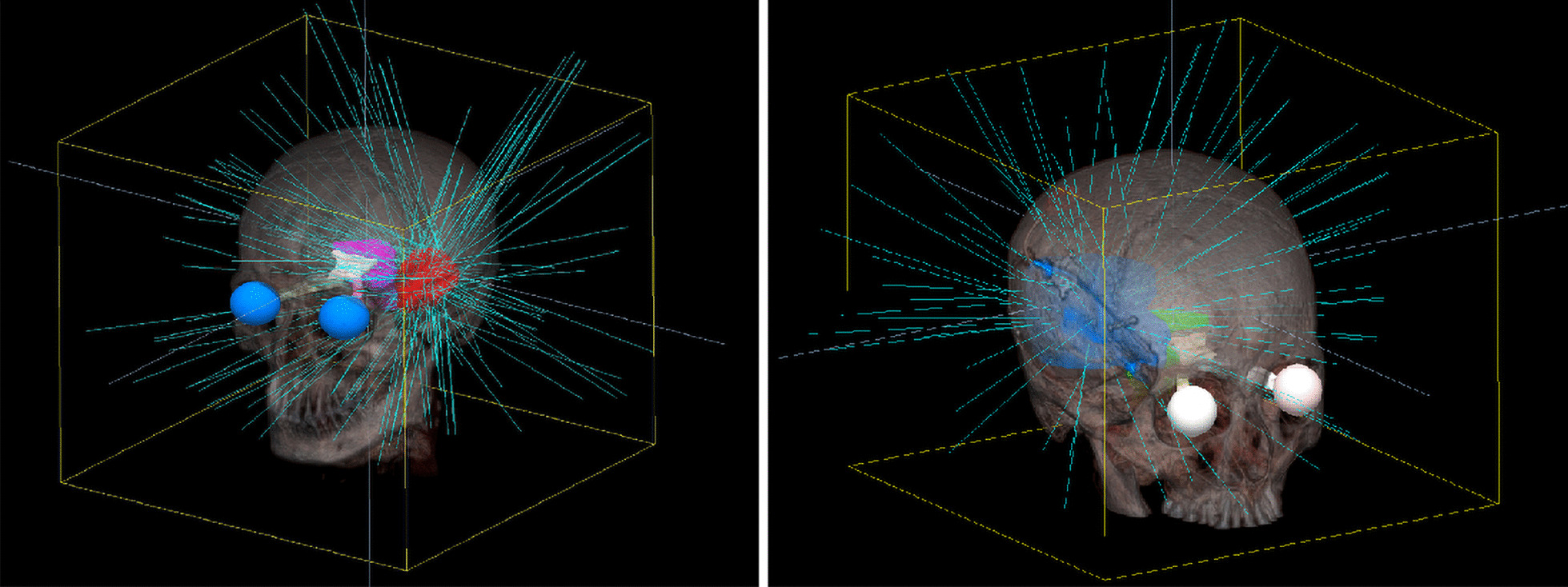
Visualization of beam distribution and structures in CK plans of unilateral tumor in temporal lobe adjacent to brainstem showing beam sets avoiding the intersection of optical apparatus

For the dose quality of protecting OARs, a double-paired significance t-test was supplemented to find that CK plans had a higher mean dose than HA plans, with p values less than 0.05, except for the left optic nerve (p = 0.09) and left eyeball (p = 0.08). There are cases in which CK showed better protection of the optical pathways. Therefore, we investigated the beam distribution and structure reconstruction images and found that the unilateral tumors in these cases were mostly located in the temporal lobe and in close proximity to the brainstem, as shown in Fig. 9. Allowing for 6 degrees of freedom, CyberKnife with its robotic arm might be more capable of avoiding the beam intersection of the brainstem and optical apparatus when all of the organs and tumors are located in the same axial slices.

**Fig. 10:**
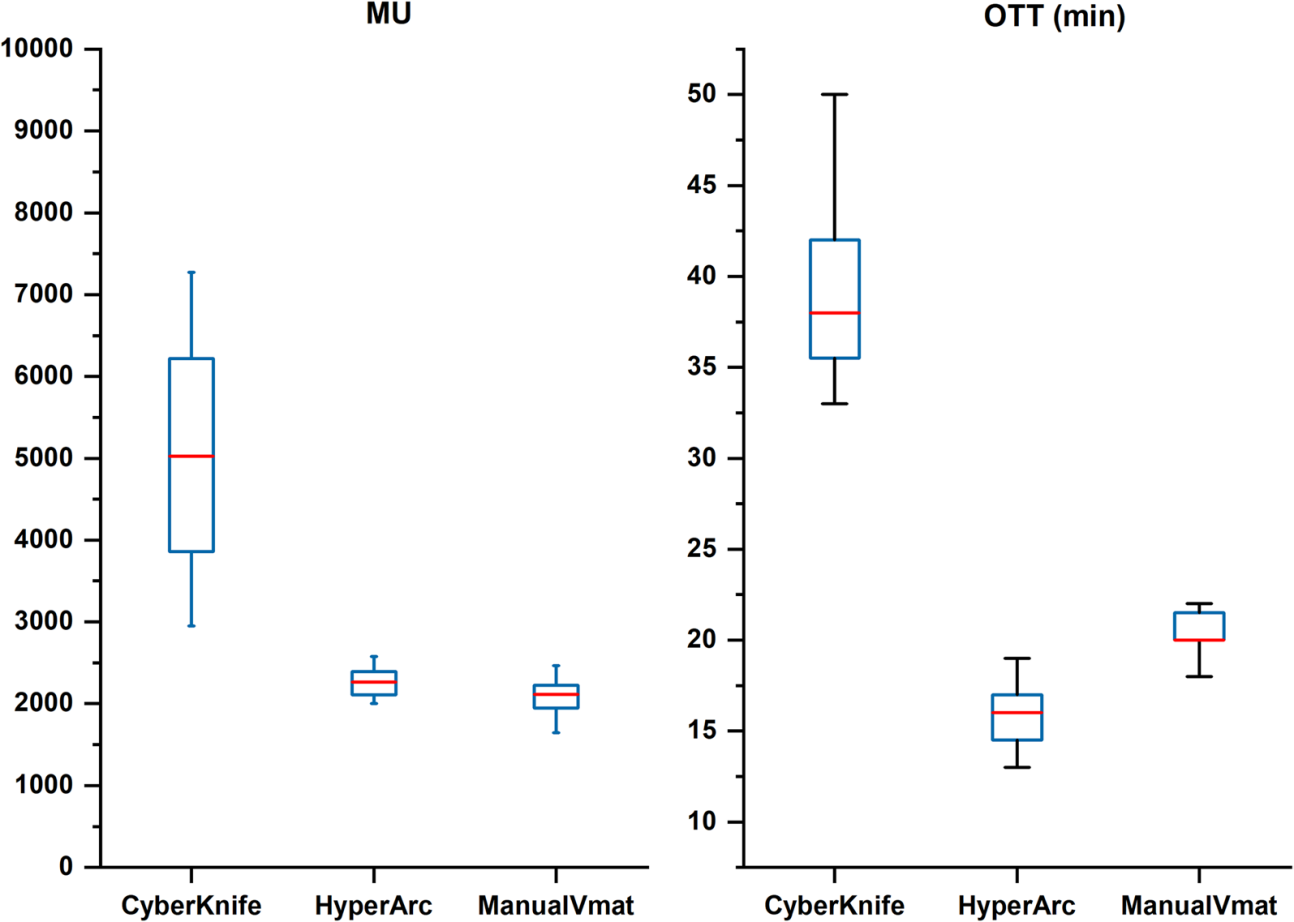
Boxplots of monitor units and overall treatment time of three techniques showed HA has a dramatic reduction in OTT

Since all of the CK plans were clinically delivered, the overall treatment time (OTT) was reduced to less than 40 min to satisfy the treatment requirement. For linac-based plans, OTT was estimated by assuming 3 min for initial patient setup plus 4 min CBCT-guided setup correction through our center’s experience, adding the beam-on time estimated in TPS. In addition, the time of couch rotation in each arc was set to 1 or 2 min for the automatic HA plans and manual VMAT plans (executed in-room). Even though the setup and correction for HA was complicated with a specific fixation device and had more MUs compared with manual VMAT, the OTT reduction of this automatic technique is still dramatic for the entire session, which can make the patient more comfortable and enhance the cost-effectiveness for the health care system, as shown in Fig. 10.

Beyond that, given the similar dosimetric quality of HA and manual VMAT, the results showed that HyperArc with 4 arcs might be sufficient in SRT for a single target. In addition, with its dry run and other automatic functions, HyperArc saves much time in planning and treatment processes and offers great convenience for medical dosimetrists and therapists.

We have already identified several points to improve the power of our future studies in this field, to increase the resulting impact for clinical medical physics. We found that the contoured structures in different TPSs with different resolutions showed a slight difference in volume calculation, which might interfere with the results of the statistical analysis, and the couch structure should be inserted into the CK plans to achieve more reliable results. More cases might be enrolled to pass the significance test when comparing normal tissue irradiation. In future studies, the specific relationship between the dosimetric quality of different techniques with the tumor location and size will be investigated, and M6 CK with Incise™ MLC will also be introduced to find the difference with fix or iris cones. Further clinical trials should be conducted, for example, retrospective analyses of patients’ OS and recurrence intervals with different treatment plans.

## Conclusions

In this study, we compared the dosimetric parameters of boosting HFRT with the novel HyperArc technique to traditional SRT techniques (like CyberKnife and conventional VMAT) for treating solitary GBM. The results of our study suggest that, from a dosimetric point of view, HyperArc was comparable and a promising alternative to the time-consuming CyberKnife treatment. With a higher CI, lower GI, and reduction of OTT, our findings indicated that HyperArc has good potential as boost therapy in the treatment of GBM, especially for lesions with a larger volume.

## Data Availability

The datasets used during the current study are available from the corresponding author on reasonable request.

## Abbreviations

BED: Biological effective dose
CI: Conformity index
CNS: Central nervous system
CTV: Clinical target volume
FFF: Flattening filter free
GBM: Glioblastoma multiforme
GI: Gradient index
GTV: Gross tumor volume
HFRT: Hypo-fractionated radiotherapy
HGG: High Grade Gliomas
HI: Homogeneity index
IRB: Institutional review board
MLC: Multi-leaf collimator
NT: Normal tissue
OARs: Organs at risk
OS: Overall survival
OTT: Overall treatment time
PIV: Prescription isodose volume
PTV: Planning target volume
SRS: Stereotactic radiosurgery
SRT: Stereotactic radiotherapy
TPS: Treatment planning system
TV: Target volume
